# Tibial Loading Increases Osteogenic Gene Expression and Cortical Bone Volume in Mature and Middle-Aged Mice

**DOI:** 10.1371/journal.pone.0034980

**Published:** 2012-04-13

**Authors:** Matthew J. Silva, Michael D. Brodt, Michelle A. Lynch, Abby L. Stephens, Daniel J. Wood, Roberto Civitelli

**Affiliations:** 1 Department of Orthopaedic Surgery, Washington University, St. Louis, Missouri, United States of America; 2 Department of Biomedical Engineering, Washington University, St. Louis, Missouri, United States of America; 3 Department of Internal Medicine, Washington University, St. Louis, Missouri, United States of America; Oklahoma State University, United States of America

## Abstract

There are conflicting data on whether age reduces the response of the skeleton to mechanical stimuli. We examined this question in female BALB/c mice of different ages, ranging from young to middle-aged (2, 4, 7, 12 months). We first assessed markers of bone turnover in control (non-loaded) mice. Serum osteocalcin and CTX declined significantly from 2 to 4 months (p<0.001). There were similar age-related declines in tibial mRNA expression of osteoblast- and osteoclast-related genes, most notably in late osteoblast/matrix genes. For example, *Col1a1* expression declined 90% from 2 to 7 months (p<0.001). We then assessed tibial responses to mechanical loading using age-specific forces to produce similar peak strains (−1300 µε endocortical; −2350 µε periosteal). Axial tibial compression was applied to the right leg for 60 cycles/day on alternate days for 1 or 6 weeks. qPCR after 1 week revealed no effect of loading in young (2-month) mice, but significant increases in osteoblast/matrix genes in older mice. For example, in 12-month old mice *Col1a1* was increased 6-fold in loaded tibias vs. controls (p = 0.001). In vivo microCT after 6 weeks revealed that loaded tibias in each age group had greater cortical bone volume (BV) than contralateral control tibias (p<0.05), due to relative periosteal expansion. The loading-induced increase in cortical BV was greatest in 4-month old mice (+13%; p<0.05 vs. other ages). In summary, non-loaded female BALB/c mice exhibit an age-related decline in measures related to bone formation. Yet when subjected to tibial compression, mice from 2–12 months have an increase in cortical bone volume. Older mice respond with an upregulation of osteoblast/matrix genes, which increase to levels comparable to young mice. We conclude that mechanical loading of the tibia is anabolic for cortical bone in young and middle-aged female BALB/c mice.

## Introduction

Mechanical loading is a powerful anabolic stimulus for bone. Methods to deliver increased mechanical loading to the skeleton represent a non-pharmacological strategy with potential to treat age-related osteoporosis [1]. For this strategy to be effective, the ability of the skeleton to respond to mechanical stimuli must persist with aging.

There is a lack of consensus on skeletal mechanoresponsiveness and aging. Exercise studies of young and aged rodents have demonstrated either reduced responsiveness in aged animals [2], [3], no difference between ages [4], [5], [6], or enhanced responsiveness in aged animals [7], [8]. Several studies that used extrinsic loading (e.g., tibial bending) reported reduced cortical responsiveness in aged turkeys [9], rats [10] and mice [11] compared to younger animals. In contrast, we recently reported no loss of cortical bone responsiveness in aged (22 month) mice compared to young-adult (7 month) mice subjected to 1 week of axial tibial compression [12].

The studies cited above on mechanoresponsiveness and aging focused on changes in bone mass or bone formation rate. A number of recent studies have described upregulation of osteogenic genes following loading in young animals [13], [14], [15]. To date there have been no reports on whether age affects loading-induced changes in expression of genes related to bone formation. Studies at the molecular level may clarify the role, if any, that age plays in the response of the skeleton to mechanical loading.

Our objective was to follow up on our previous study that used axial tibial compression in young-adult and aged mice [12], and to focus on short-term molecular and longer-term structural effects. Because we observed no decline in responsiveness from 7 to 22 months, we asked if a decline might occur earlier in the lifespan. In addition, we asked if age affected the upregulation of osteogenic genes following loading. Therefore, we compared responses to axial tibial compression in mice of different ages, ranging from young to middle-aged (2–12 months). We applied age-specific forces to produce similar values of peak strain. We assessed markers of bone turnover in non-loaded control mice, and then assessed bone responses to loading using molecular (quantitative RT-PCR) and structural (in vivo microCT) outcomes.

## Results

### Markers of bone formation are diminished with maturation

**Figure 1:**
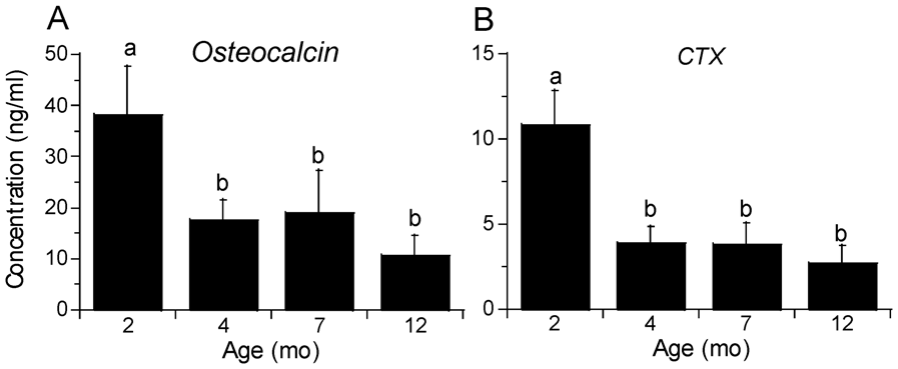
Systemic bone turnover decreases with age. Serum was collected from baseline control (non-loaded) mice of four different ages, and indices of bone turnover were measured by ELISA. (a) Osteocalcin, a marker of bone formation, and (b) carboxyl-terminal collagen cross-links (CTX), a marker of bone resorption, declined significantly with age. (n = 8; mean ± SD; a,b: groups with no common letters are significantly different, p<0.05).

Based on cross-sectional analysis of control mice at different ages, serum markers of bone formation (osteocalcin) and resorption (CTX) declined significantly from 2 to 4 months age (p<0.001; Figure 1). Osteocalcin further declined from 7 to 12 months age, although this difference did not reach statistical significance (p<0.10). CTX did not change after 4 months.

**Figure 2:**
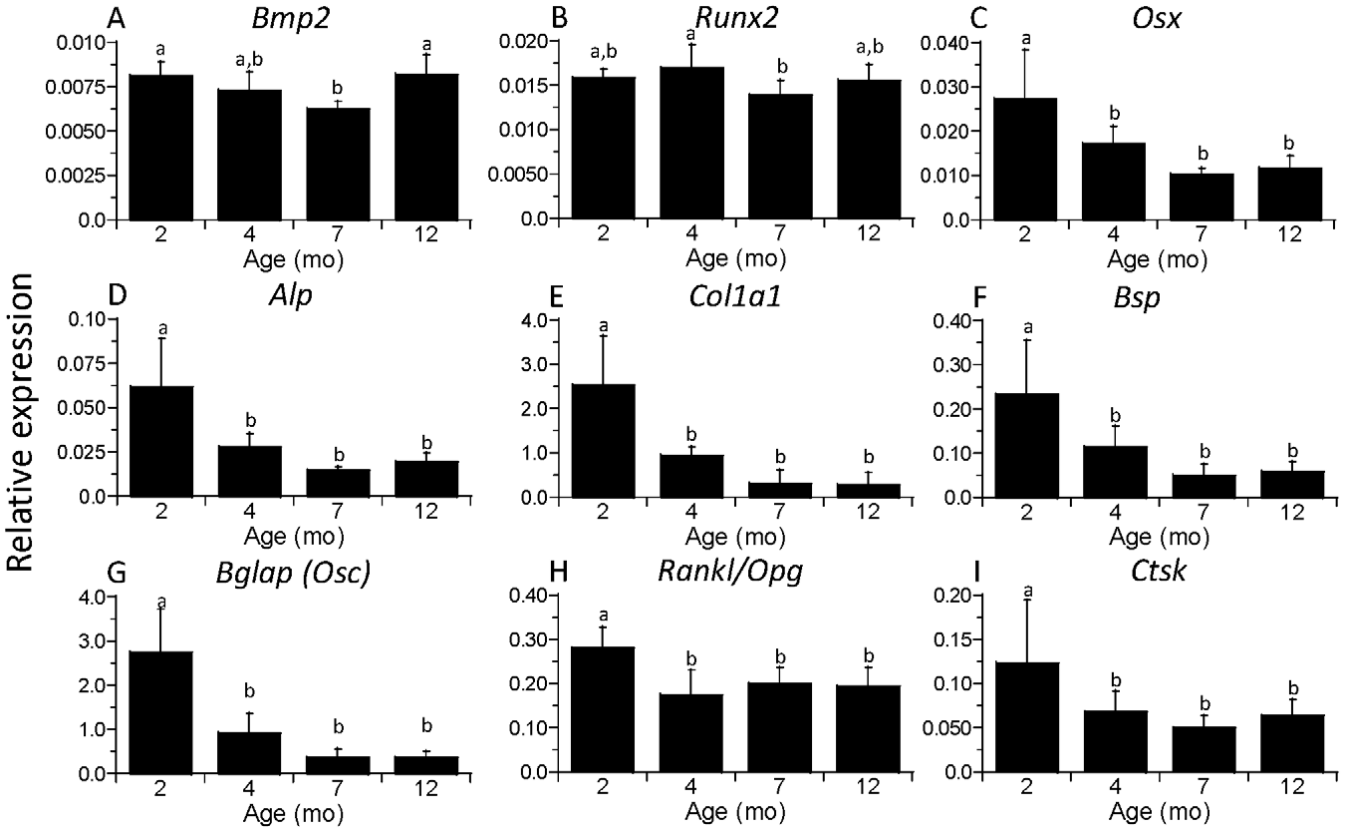
Expression of bone genes is age dependent. mRNA was isolated from the tibia of baseline, non-loaded control mice of four different ages, quantified by real-time RT-PCR and expressed relative to the housekeeping gene *cyclophilin*. Expression of most osteoblast- and matrix-related genes was age-dependent, with significant declines from 2 to 4 months. Expression of osteoclast-related genes also declined from 2 to 4 months but not thereafter. (n = 7–8; mean ± SD; a,b: groups with no common letters are significantly different, p<0.05).

Similarly, analysis of tibial mRNA revealed declines from 2 to 4 months age in the expression of many osteoblast- and osteoclast-related genes (Figure 2). The clearest pattern was in the osteoblast and matrix genes *Osx*, *Alp*, *Col1a1*, *Bsp* and *Bglap*, which declined from 35–65% from 2 to 4 months (p<0.01). Expression of *Osx*, *Col1a1* and *Bglap* declined from 4 to 7 months, although these differences did not reach statistical significance (p<0.10); there were no further changes from 7 to 12 months. The *Rankl*/*Opg* ratio, an important factor for osteoclastogenesis, and the osteoclast activity gene *Ctsk* also declined from 2 to 4 months (p<0.01), but not thereafter. In contrast, the bone morphogen *Bmp2* and the early osteoblast transcription factor *Runx2* did not change from 2 to 4 months age. *Bmp2* did decrease modestly from 2 to 7 months (p<0.05), but then increased from 7 to12 months (p<0.001), the only gene that showed such a pattern. *Runx2* showed no clear pattern with age. In summary, nearly all systemic and local measures of bone turnover declined significantly from 2 to 4 months age; there were only marginal changes after 4 months.

### 1 week of tibial loading increases expression of bone formation genes in older mice

**Figure 3:**
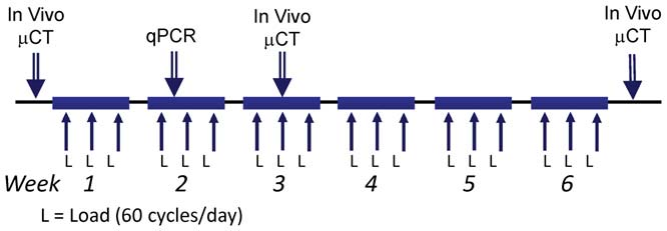
Study timeline. Mice were loaded 3 days/wk.

**Figure 4:**
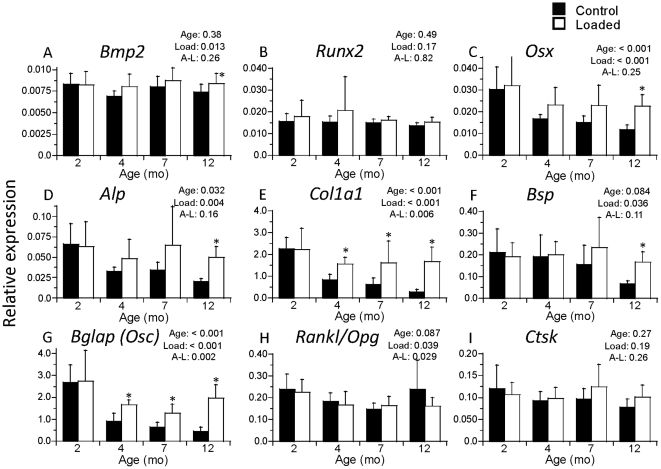
Mechanical loading upregulates bone genes in older mice. Bilateral tibias (left, Control; right, Loaded) were harvested after 1 week of unilateral mechanical loading. mRNA was isolated, quantified by real-time RT-PCR, normalized to the housekeeping gene *cyclophilin*, and then expressed as fold difference of loaded relative to control. There were no significant differences between loaded and control limbs at 2 months age. However, with increasing age there was an increase in relative expression of osteoblast and bone matrix genes in loaded versus control limbs. (n = 7–8; mean ± SD; p-values for Age, Load and Age-Load interaction are from 2-factor ANOVA; * loaded different from control, p<0.05).

The right legs of mice were subjected to tibial compression on alternate days for 1 week followed by gene expression analysis of right (loaded) and left (contralateral control) tibias (Figure 3). Age-specific forces were applied to produce similar peak strains (approx. −1300 µε endocortical; −2350 µε periosteal). Expression of seven of nine genes examined (all but *Runx2* and *Ctsk*) was higher in loaded tibias vs. controls (p<0.05; Figure 4). Expression of five of these seven genes depended significantly on age or had a significant age-load interaction (p<0.05). Specifically, at 2 months age, there were no significant differences between loaded and control bones; at 4 and 7 months, expression of *Col1a1* and *Bglap* were greater in loaded than control bones (p<0.05); at 12 months, *Osx*, *Alp*, *Col1a1*, *Bsp*, and *Bglap* were greater in loaded than control bones (p<0.005). The largest differences between loaded and control occurred in the 12-month group for the osteoblast/matrix genes *Col1a1* (up 6.2 fold) and *Bglap* (up 4.4 fold).

Comparisons of gene expression in contralateral control bones from loaded mice of different ages revealed similar findings as for the baseline controls, i.e., the expression of *Osx*, *Alp*, *Col1a1* and *Bglap* were lower at older ages (p<0.05; Figure 4). For example, at 12 months the expression of *Col1a1* was 8.4-fold less than at 2 months, while the expression of *Bglap* was 5.9-fold less. In contrast, in loaded tibias the relative expression of genes did not vary significantly with age, with the exception of *Bglap* which was 2.2-fold lower at 7 months than at 2 months (p = 0.011). Thus, age-related differences in expression of osteoblast/matrix genes that were observed in control bones were largely not observed in bones after 1 week of tibial compression.

### 6 weeks of tibial compression increases cortical bone volume at all ages

**Figure 5:**
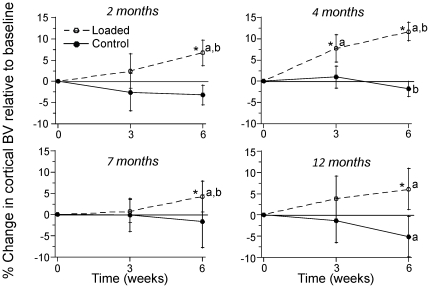
Mechanical loading increases cortical bone volume in all age groups. Cortical bone volume (BV) was assessed at the mid-diaphysis of loaded and contralateral control limbs using in vivo microCT at study baseline (0 weeks), middle (3 weeks) and end (6 weeks). Percent change was computed relative to the value at baseline. Cortical BV increased regardless of age in legs subjected to axial tibial compression. In each age group, significant temporal increases were observed in loaded tibias but not in contralateral controls. (n = 7–9; mean ± SD; * loaded different from control; a: different from 0 weeks; b: different from 3 weeks, p<0.05).

The right legs of mice were subjected to tibial compression on alternate days for 6 weeks (Figure 3). Again, age-specific forces were applied to produce similar peak strains (approx. −1300 µε endocortical; −2350 µε periosteal). MicroCT scans of the mid-diaphysis at the 6-week timepoint revealed that loaded tibias in each age group had significantly greater cortical bone volume (BV) than contralateral control tibias (p<0.05; Figure 5; Table 1). This difference between loaded and control tibias was due to a significant increase in cortical BV with time in loaded tibias from each age group, while BV in control tibias either decreased slightly (4, 12 month groups) or did not change (2, 7 month groups). The increase in cortical BV of loaded tibias from baseline to 6 weeks was significantly greater in the 4-month group (+13%) compared to other age groups (p<0.05). Analysis of medullary volume (MV) and total volume (TV) indicated that the mechanism of increase in cortical BV was increased TV (i.e., periosteal expansion) in the 4, 7 and 12 month groups, and prevention of decreased TV (i.e., periosteal maintenance) in the 2 month group. Thus, tibial compression increased cortical bone volume by relative expansion of the periosteal margin in all age groups.

**Table 1:** Cortical bone volume and density from in vivo microCT scans at baseline, middle and end of 6-week loading period.

Outcome	Age	Left (Control)			Right (Loaded)		
		0 wk	3 wk	6 wk	0 wk	3 wk	6 wk
Bone Volume (mm^3^)	2 month (n = 7)	0.419±0.015	0.408±0.023	0.405±0.013	0.411*±0.012	0.420±0.019	0.438* a b±0.019
Medullary Volume (mm^3^)		0.278±0.016	0.257^a^±0.017	0.216^a^ ^b^±0.007	0.291±0.036	0.251^a^±0.030	0.238* a±0.024
Total Volume (mm^3^)		0.697±0.023	0.666^a^±0.031	0.622^a^ ^b^±0.016	0.702±0.045	0.672±0.036	0.677*±0.040
TMD (mg HA/cm^3^)		1040±16	1078^a^±27	1125^a^ ^b^±12	1037±27	1084^a^±16	1126^a^ ^b^±6
Bone Volume (mm^3^)	4 month (n = 8)	0.457±0.032	0.461±0.032	0.448^b^±0.033	0.455±0.030	0.489* a±0.019	0.508* a b±0.031
Medullary Volume (mm^3^)		0.232±0.030	0.234±0.032	0.226±0.035	0.223±0.037	0.239±0.037	0.250^a^±0.042
Total Volume (mm^3^)		0.688±0.058	0.695±0.062	0.674±0.064	0.678±0.062	0.728^a^±0.050	0.758* a b±0.069
TMD (mg HA/cm^3^)		1123±17	1145^a^±16	1167^a^ ^b^±12	1136±13	1151^a^±14	1175^a^ ^b^±12
Bone Volume (mm^3^)	7 month (n = 9)	0.466±0.029	0.465±0.020	0.458±0.030	0.466±0.036	0.469±0.026	0.485* a b±0.026
Medullary Volume (mm^3^)		0.202±0.031	0.200±0.022	0.198±0.023	0.212±0.034	0.220*±0.032	0.219*±0.028
Total Volume (mm^3^)		0.668±0.056	0.665±0.037	0.656±0.040	0.678±0.061	0.689±0.053	0.705* a b±0.049
TMD (mg HA/cm^3^)		1164±12	1173±17	1162±42	1170±17	1184* a±10	1184^a^±16
Bone Volume (mm^3^)	12 month (n = 7)	0.474±0.035	0.467±0.031	0.449^a^±0.022	0.471±0.017	0.488±0.020	0.499* a±0.016
Medullary Volume (mm^3^)		0.201±0.012	0.213^a^±0.019	0.215^a^±0.023	0.205±0.014	0.216±0.017	0.228^a^±0.010
Total Volume (mm^3^)		0.674±0.042	0.680±0.035	0.664±0.041	0.676±0.025	0.704±0.033	0.727* a±0.022
TMD (mg HA/cm^3^)		1195±16	1183±20	1182±26	1198±10	1209* a±10	1206*±8

mean ± SD;

* Right different from Left;

a : different from 0 wk;

b : different from 3 wk; p<0.05.

### 6 weeks of tibial compression decreases trabecular BV/TV in 4, 7 and 12 month mice

**Figure 6:**
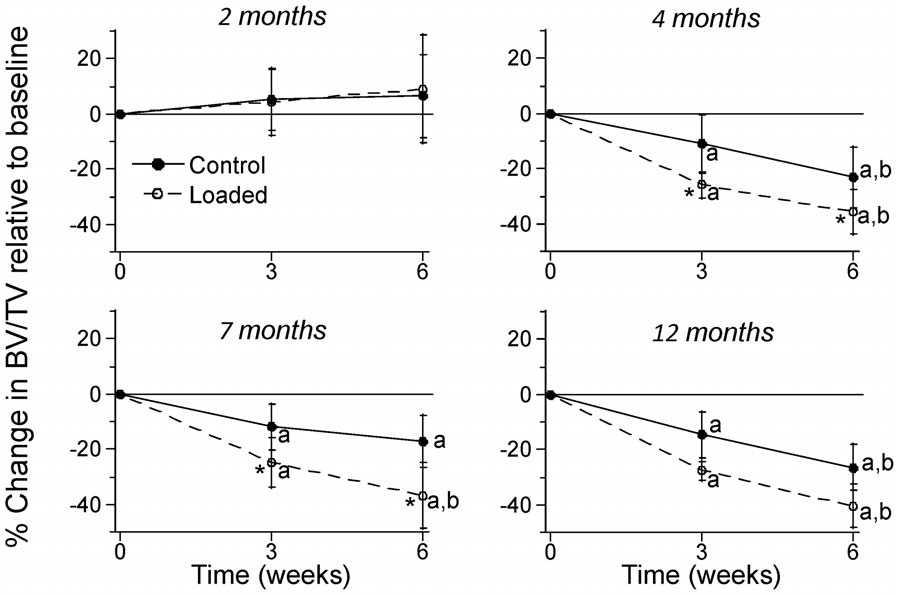
Mechanical loading decreases trabecular bone volume in older mice. Trabecular bone volume fraction (BV/TV) decreased in legs of 4, 7 and 12 month old mice subjected to axial tibial compression. BV/TV was assessed in the proximal tibias of loaded and contralateral control limbs using in vivo microCT at the study baseline (0 weeks), middle (3 weeks) and end (6 weeks). Percent change in BV/TV was computed relative to the value at baseline. Significant temporal decreases were observed in mice aged 4, 7 and 12 months, with greater declines in loaded limbs than controls. (n = 7–9; mean ± SD; * loaded different from control; a: change in BV/TV is different from 0 weeks; b: change in BV/TV is different from 3 weeks, p<0.05).

MicroCT scans of the proximal tibial metaphysis revealed that loaded tibias in the 4- and 7-month age groups had lower trabecular bone volume fraction (BV/TV) than control tibias at the 6-week timepoint (p<0.05; Figure 6; Table 2). In the 4–12 month age groups, both loaded and control tibias had reduced BV/TV with time (p<0.05), but loaded tibias lost significantly more BV/TV than controls (p<0.05). There were no changes with time in tibial BV/TV of 2-month old mice. Thus, in contrast to its anabolic effect on diaphyseal cortical bone, tibial compression had a catabolic effect on metaphyseal trabecular bone in older mice.

**Table 2:** Trabecular bone microstructure and density from in vivo microCT scans at baseline, middle and end of 6-week loading period.

Outcome	Age	Left (Control)			Right (Loaded)		
		0 wk	3 wk	6 wk	0 wk	3 wk	6 wk
BV/TV (mm^3^/mm^3^)	2 month (n = 8)	0.233±0.034	0.245±0.041	0.248±0.044	0.229±0.031	0.238±0.034	0.250±0.057
Tb.Th (mm)		0.060±0.003	0.063^a^±0.004	0.064^a^±0.004	0.057*±0.003	0.062^a^±0.004	0.067* a b±0.003
Tb.N (1/mm)		5.8±0.5	5.8±0.4	5.5^a^ ^b^±0.5	5.9±0.5	5.8±0.3	5.5±0.7
vBMD (mg HA/cm^3^)		212±19	221±25	222±27	208±19	217±22	226±38
BV/TV (mm^3^/mm^3^)	4 month (n = 8)	0.294±0.041	0.262^a^±0.049	0.227^a^ ^b^±0.052	0.291±0.027	0.216* a±0.027	0.189* a b±0.038
Tb.Th (mm)		0.069±0.003	0.068±0.003	0.068±0.004	0.066*±0.003	0.059* a±0.003	0.063* b±0.005
Tb.N (1/mm)		6.4±0.7	5.8^a^±0.6	5.0^a^ ^b^±0.7	6.3±0.3	5.4^a^±0.2	5.0^a^ ^b^±0.3
vBMD (mg HA/cm^3^)		250±21	232^a^±30	214^a^ ^b^±34	253±16	205* a±16	189* a b±24
BV/TV (mm^3^/mm^3^)	7 month (n = 9)	0.231±0.019	0.203^a^±0.029	0.191^a^±0.031	0.237±0.023	0.178* a±0.029	0.149* a b±0.029
Tb.Th (mm)		0.067±0.002	0.066±0.006	0.066±0.006	0.068±0.004	0.060±0.006* a	0.059* a±0.003
Tb.N (1/mm)		4.9±0.3	4.6^a^±0.4	4.3^a^ ^b^±0.3	4.8±0.5	4.5±0.4	4.2^a^±0.4
vBMD (mg HA/cm^3^)		215±11	198^a^±18	189^a^±21	220±15	179* a±19	158* a b±19
BV/TV (mm^3^/mm^3^)	12 month (n = 7)	0.220±0.032	0.189^a^±0.039	0.163^a^ ^b^±0.037	0.277±0.146	0.202^a^±0.111	0.172^a^ ^b^±0.116
Tb.Th (mm)		0.073±0.004	0.070±0.002	0.070±0.005	0.089±0.044	0.073±0.028	0.070^a^±0.022
Tb.N (1/mm)		4.0±0.7	4.0±0.4	3.7^a^ ^b^±0.4	4.2±0.8	3.9^a^±0.6	3.8^a^±0.7
vBMD (mg HA/cm^3^)		207±20	187^a^±27	168^a^ ^b^±26	250±107	196^a^±79	173^a^±77

mean ± SD;

* Right different from Left;

a : different from 0 wk 0;

b : different from 3 wk; p<0.05.

## Discussion

We assessed the response to skeletal loading in female BALB/c mice in the age range from youth to middle age. We determined that systemic and local markers of bone turnover in non-loaded control animals decline significantly with maturation (i.e., from 2 to 4 months); with further aging there were only non-significant trends for modest decline in bone formation markers. One week of skeletal loading by axial tibial compression increased the relative expression of osteoblast/matrix genes in older mice, offsetting some of the age-related decline. Six weeks of loading led to increased cortical bone volume by relative periosteal expansion in all age groups (2, 4, 7, 12 months). In contrast, 6 weeks of loading decreased trabecular bone volume in 4–12 month old mice. Taken together these findings indicate that, despite an age-related decline in expression of some osteogenic genes, skeletal loading can increase cortical bone volume in mice from youth to middle age.

The age-related declines in systemic and local markers of bone formation that we find in female BALB/c mice are consistent with the limited available data from other inbred mouse strains. We observed a sharp decline in serum osteocalcin from 2 to 4 months and a trend for further decline from 7 to 12 months. Others have studied C57Bl/6 and C3H/He mice and reported that serum osteocalcin declines from 1 to 3 [16] and 3 to 12 months [17], and serum alkaline phosphatase declines from 1 to 8 months age [18]. At the bone level, we also observed significant declines in expression of osteoblast and bone matrix genes from 2 to 4 months age, with trends for further decline from 4 to 7 months. Others have reported significant decreases in osteocalcin [19] and alkaline phosphatase [18] activity in mouse bones from 1 to 3 months age. In addition, from 1 to 6 to 24 months age, tibial *Col1a1* and *Alp* expression declined in C57Bl/6 mice [20]. We previously noted a modest decline in cortical and trabecular bone formation rates from 7 to 22 months age in BALB/c mice [21]. (Mice in that study were subjected to sham or whole-body vibration loading; the age-related decline in bone formation rates was noted at the periosteal surface of the tibial diaphysis, and the trabecular bone in the proximal tibia and is based on data from all groups pooled, as there was no effect of vibrational loading.) Similarly, Ferguson et al. reported that cortical bone formation in C57Bl/6 mice declines sharply from 1 to 5 months, and beyond 5 months age there is no consistent fluorochrome labeling [22]. In summary, the available data indicate that bone formation in mice declines significantly from youth to middle age.

We detected significant increases in expression of osteoblast and bone matrix genes in loaded compared to control bones 1 week after the start of loading, most notably in older mice. This finding is consistent with reports describing increases in similar genes at 3–12 day timepoints in other loading models in mice [23] and rats [13], [14]. Somewhat unexpectedly, we did not observe increased expression in loaded bones from 2-month old mice, whereas the greatest loading effects were observed in 12-month old mice. Others have reported that tibial bending in 2-month old C57Bl/6 mice led to increased expression of osteoblast-related genes [23], although the mechanical strain applied in that study was much greater than the current study (tensile gage-site strain ∼3500 vs. ∼1500 µε). One possible reason for the lack of a gene expression response to loading in 2-month old mice is because the baseline levels of expression were already high. Perhaps an even greater stimulus is required to enhance gene expression in bones from young animals or under other conditions of high baseline activity. Importantly, in older mice loading increased the expression of genes related to bone formation, effectively offsetting much of their age-related decline.

Mechanical loading significantly increased cortical bone volume at all ages. The greatest effect occurred at 4-months, where the increase in cortical BV was approximately twice as great as at other ages (Fig. 5). This contrasts the gene expression results which indicated that the 12-month group was the most responsive to loading. The reason for this inconsistency is unclear. It may simply reflect the different timepoints for gene expression (1 week) versus cortical bone volume (3 and 6 weeks) measurements. It is also possible that loading triggers non-transcriptional events that influence bone accrual. Regardless, our results suggest that the magnitude of the gene expression response at one particular time may not be a good predictor of the eventual increase in bone mass.

We observed some bone loss in control limbs, which suggests a negative systemic effect of loading. Cortical BV declined in non-loaded limbs from 4- and 12-month groups, while trabecular bone declined in non-loaded limbs from 4-, 7- and 12-month groups. Because we did not include an age-matched control group we cannot determine if this loss was simply the result of normal changes over the 6-week duration of our study. Data from non-loaded female BALB/c mice obtained at different ages indicate that cortical bone is not normally lost during growth and maturation, although tibial trabecular BV/TV does decline progressively after 4-months age [24]. Ongoing studies are addressing the issue of systemic effects in our loading protocol.

Our tibial compression loading protocol was not anabolic for *trabecular* bone at the proximal tibial metaphysis, despite being anabolic for *cortical* bone at the mid-diaphysis. Loading did not cause a change in trabecular BV/TV in 2-month old mice, and caused a significant decrease in 4-, 7- and 12-month mice. This finding confirms our recent results using the same loading protocol in 7- and 22-month old male BALB/c mice [12]. In contrast, there are several reports of increased trabecular BV/TV after tibial compression in growing [25], [26] and mature C57Bl/6 mice [27], [28]. We rule out trabecular microfracture as a cause of bone loss in our study, as microCT scans from the current study and histological examination of samples in a previous study revealed no evidence of microfracture [12]. We can not rule out more subtle forms of microdamage, as we have not stained samples to look for microcracks or diffuse damage. Recent unpublished data from our lab indicate that mouse strain and loading history are factors in the loss of trabecular bone we observed. Briefly, C57Bl/6 mice subjected to the same loading history used in the current study did not lose trabecular bone, indicating that different inbred strains respond differently, perhaps due to differences in baseline trabecular BV/TV (BALB/c ∼0.25; C57Bl/6 ∼0.15 [25], [26], [28]). Moreover, when we used a loading protocol that has been used by other groups (1200 cycles/day; 4 Hz) [26], [27], we observed increased trabecular bone volume. Additional studies are needed to confirm these preliminary findings and determine their underlying basis.

Returning to our main question: Does the aging skeleton lose its ability to respond to mechanical loading? Our current view is that there is a partial, but not complete loss of cortical mechanoresponsiveness with aging. The 4-month old mice here added the greatest amount of cortical bone, suggesting that there is an age when the skeleton most efficiently converts mechanical stimuli into increased bone mass. In support of this view, several previous studies found that loading protocols that were potently anabolic in younger animals stimulated little to no response in older animals [9], [10], [11]. On the other hand, a number of studies (including the present one) have found that declines in mechanoresponsiveness with aging are more modest and that aged animals maintain an ability to respond robustly to loading. Kesavan et al. [23] reported no difference in anabolic responses to tibial bending between 2-, 4- and 9-month old mice (both C57Bl/6 and C3H/He). In a recent study, we observed that tibial compression stimulated equal or greater cortical bone responses in 22-month old BALB/c mice compared to 7-month old mice [12]. Moreover, several (though not all [2], [3]) exercise studies in rodents reported that the skeletal benefits of exercise are not limited by age [4], [5], [6], [7], [8]. For example, 12 weeks of daily treadmill running [8] and 8 weeks of daily jumping [5] each produced significant increases in bone mass in 2-year old rats. Critically, there is no proven mechanistic basis to support a loss of mechanoresponsiveness with age. On the one hand, there are reports of decreased osteocyte number with aging [29], [30], which might lead to diminished mechanotransduction. On the other hand, in vitro studies have reported little or no cell autonomous decline in mechanoresponsiveness with aging [31], [32], [33]. In summary, there is increasing evidence that the aging skeleton maintains its ability to respond positively to loading.

One strength of our study was the use of longitudinal assessment of bone using in vivo microCT. Because 6 weeks is a relatively long duration for a loading study, we anticipated changes in bone structure would be detectable by microCT. For this reason we elected not to use histomorphometry to measure traditional dynamic indices of bone formation. This limits our ability to make strong conclusions about endocortical vs. periosteal responses. Although we do not express our data as rates of volume change, this could be done using any two timepoints from the microCT data (Tables 1, 2). Also, because we expressed changes relative to baseline (Figures 5,6) we are able to state whether there was bone loss or gain, not just a relative benefit of loading. Therefore, we chose not to report relative differences (loaded – control); data analysis based on relative differences did not lead to any change in our conclusions (data not shown).

Our study had several limitations. First, we analyzed only a single timepoint for gene expression and focused on osteoblast/matrix genes rather than early response genes or signaling pathways. Peak rates of bone formation occur 5–8 days after a single bout of loading [34]. Thus, the 1-week timepoint was selected to reflect the cumulative effects of the first three loading sessions, when expression of osteoblast/matrix genes should be relatively high. Analysis of bones after 6 weeks of loading showed few differences in expression between loaded and control tibias (data not shown), suggesting that the tibia had accommodated to the loading stimulus. A second limitation is the homogenization of the entire tibia (bone plus marrow) for qPCR analysis. This approach does not allow for evaluation of local differences. Thus, our qPCR analysis does not clarify the differences in the metaphyseal and diaphyseal sites seen by microCT. Another limitation is the use of a single loading force for each age group, chosen to provide an equivalent local strain stimulus. This “strain matching” strategy is standard for animal studies of mechanoresponsiveness, although it may not reflect the overall mechanical stimulus at the organ level. One way to offset this limitation in future studies is to use a “force matching” approach in addition to “strain matching”. Based on the strain approach, we determined that the 7-month group required a lower force than the 4- or 12-month groups to produce an equivalent local strain magnitude (Table 3). Although this is not what we expected, Lynch et al. observed a similar phenomenon in C57Bl/6 mice [26], [27]: they applied 11.5 N force to produce 1200 microstrain at the tibial mid-shaft in 2.5-month old mice, but applied only 5.9 N to produce the same strain in 6-month mice. We note that the 7-month group in our study had the smallest value of total volume (TV; Table 1) which may have contributed to the lower overall stiffness. We further suggest that age-related differences in curvature along the length of the tibia alter the force-strain relationships, although we did not assess curvature in this study. Another limitation related to loading was the use of a constant force rate (48 N/sec), which resulted in different strain rates between groups. The estimated strain rates (based on a peak of 1300 µε) would have ranged from 5700 to 8300 µε/s. These differences in strain rates may have contributed to variability in response, although we note that the most responsive group (4 month) had the second lowest strain rate (6250 µε/s).

**Table 3:** Average strain values (microstrain, µε) for the applied forces used for in vivo tibial compression.

Age (mo)	Force (N)	Gage Site	Max Periosteal: Tension	Max Endocortical: Tension	Min Periosteal: Compression	Min Endocortical: Compression
2	−7.5	1290	1540	1080	−2200	−1340
4	−10	1340	1520	1030	−2320	−1270
7	−8.5	1480	1690	1040	−2490	−1390
12	−11	1510	1720	1050	−2520	−1390

Strains were measured at gage site; values at other sites obtained by linear interpolation of measured value based on cross-sectional geometry; shaded cells indicate target values.

In summary, there is a normal age-related decline in markers of bone formation in female BALB/c mice. Despite this decline, mice from 2–22 months [12] have an anabolic cortical response to tibial compression. Current data indicate that maturing mice (4 month) are more responsive to loading than mice at other ages. Older mice respond with a marked upregulation of osteoblast and bone matrix genes, which increase to levels comparable to young, growing mice. We conclude that mechanical loading can be anabolic for cortical bone in older mice despite lower baseline bone formation.

## Materials and Methods

### Ethics Statement

The research described in this study involving animals is in compliance with all applicable Federal regulations and University and sponsoring agency policies and procedures. The study was approved by the Washington University Animal Studies Committee (protocol number 20080258).

### Animals

Female BALB/cBy mice were purchased from the National Institute of Aging (NIA, Bethesda, MD, USA) aged mouse colony at four different ages. BALB/c mice have an intermediate bone mass compared to “low bone mass” C57Bl/6 and “high bone mass” C3H/He inbred strains [35]. They exhibit similar age-related changes in bone structure and density as C57Bl/6 mice [24], and they have an anabolic cortical bone response to tibial compression [12]. Age groups were selected to represent four life stages [36]: growing (2 month), maturing (4 month), mature (7 month), and middle-aged (12 month). After arrival mice were housed for 1–2 week before experimental use. Animals were housed 4–5 per cage with unlimited mouse food and water and were exposed to a 12∶12 hour light:dark cycle. Some mice were used as baseline controls, and others were subjected to axial tibial compression.

### Baseline controls

Serum markers of bone turnover and tibial gene expression were assessed using a set of 32 baseline control (non-loaded) mice (2, 4, 7, 12 months age; n = 8/group). Food was withdrawn 6 hr before death. Mice were anesthetized (i.p. ketamine [100 mg/kg] and xylazine [10 mg/kg]), and cardiac puncture was performed; mice died by exsanguination. Blood samples were centrifuged (8000 rpm, 5 min) and the serum stored (−80°C) until later use. The central portion (∼60–75%) of each tibia was harvested within 8 min of death, flash frozen in liquid nitrogen, and stored (−80°C) until RNA isolation.

Serum markers of bone turnover were assessed using kits. Osteocalcin (Mouse Osteocalcin EIA Kit; Biomedical Technologies Inc., Stoughton, MA, USA) was used as a marker of bone formation, while the c-terminal cross-linking telopeptide of type I collagen (CTX) (RatLaps™ EIA; Immunodiagnostics Systems Inc., Fountain Hills, AZ, USA) was used as a marker of resorption.

### Gene expression

The central portion of the tibias from both baseline control mice and 1-week loaded mice (see below) was used for quantitative gene expression by real-time reverse transcriptase polymerase chain reaction (qRT-PCR) using a previously described protocol [37]. Samples included cortical bone and marrow. Briefly, each sample was placed in a liquid nitrogen-cooled stainless steel flask along with a steel ball and shaken until pulverized (Micro-Dismembrator, B. Braun Biotech Inc.). The sample was then stabilized using TRIzol (Invitrogen, Carlsbad, CA, USA), and DNA and proteins were precipitated out of solution using chloroform (Sigma, Saint Louis, MO, USA) and phase lock gel tube (PLG-heavy, Eppendorf). The RNA was further purified using the RNeasy Mini Kit (Qiagen, Germantown MD, USA). The purity and concentration of the isolated RNA was determined using a spectrophotometer (Nanodrop 2000, Thermo Scientific, Wilmington, DE, USA). 1 µg of purified RNA was used to synthesize cDNA using random primers and superscript III reverse transcriptase (Invitrogen). qRT-PCR reactions were carried out using 20 µl of final volume and 40 cycles of denaturing and annealing/elongation (7500 Real-Time PCR System, Applied Biosystems, Foster City, CA, USA). SYBR green (Applied Biosystems) was used as a reporter agent and the cycle number to threshold (CT) was determined using the manufacturer's software. Nine genes were assessed: *Bmp2*, *Runx2*, *Osx*, *Alp*, *Col1a1*, *Bsp*, *Bglap*, *Rankl*, *Opg* and *Ctsk*. (See Table S1 for primer information.) Expression levels were normalized relative to the expression of the housekeeping gene cyclophillin (*Cyclo*). We confirmed that *Cyclo* expression did not differ between age groups or between loaded vs. control legs (p>0.05). For baseline controls, values are presented as fold-difference relative to *Cyclo* (2^−ΔCT^). For 1-week loaded tibias, values are presented as fold-difference relative to contralateral control (2^−ΔΔCT^).

### Force-strain analysis

A set of 22 mice was used to determine the axial force that produced peak tibial strains of approximately1100 µε tension and −1300 µε compression on the endocortical surface at the mid-diaphysis. This was done for each age group (2, 4, 7, 12 months age; n = 5–6/group). These values of strain were chosen to match the intermediate loading level used in our previous study, where we also used endocortical strain as our target [12]. Briefly, each mouse was euthanized by CO_2_ asphyxiation and a single element strain gage (FLK-1-11-1L, Texas Measurements, College Station, TX, USA) was applied to the antero-medial periosteal surface at the approximate site of peak tensile strain, 5 mm proximal to the distal tibio-fibular junction. The tibia was then loaded (waveform description below) to peak forces of 4 to 14 N (2 N increments) and strain was recorded. Relationships between peak force vs. peak gage-site strain were determined by linear regression. Strain values at the gage site were linearly interpolated to values at the sites of peak tension and compression on the endocortical surface based on dimensions obtained from microCT scans of the tibia with gage attached, a method we used previously [12], [38]. The compressive force values that produced the target strains were: 7.5, 10.0, 8.5 and 11.0 N for 2, 4, 7 and 12 month old mice, respectively. The measured periosteal strain, and the estimated periosteal and endocortical strains engendered by these forces are listed in Table 3.

### In vivo loading

Responses to in vivo loading were assessed in tibias from a total of 63 mice from four age groups (2, 4, 7, 12 months). Axial tibial compression is a non-invasive method to stimulate bone formation in mice [39], [40]. As described [12], mice were anesthetized (2% isofluorane) and their right lower legs subjected to axial compression for 60 cycles/day using a rest-inserted protocol (preload: 0.5 N; triangle waveform with loading/unloading rate: 48 N/sec; rest between cycles: 10 sec) applied using a materials testing system (Dynamite 8841, Instron, Norwood, MA, USA). The peak force was age-specific, as described above. Mice were loaded for 3 days/week (Mon/Wed/Fri). For gene expression by qPCR, a set of mice (n = 7–8/age group) were loaded for four sessions (Mon/Wed/Fri/Mon) and euthanized 24 h after the last loading session (Figure 3). For structural analysis by in vivo microCT, a set of mice (n = 7–9/age group) were loaded 18 times over 6 weeks (Figure 3). The left leg served as a non-loaded, contralateral control. After each loading session, mice received buprenorphine (0.1 mg/kg i.m.) and were returned to their cages and allowed unrestricted activity. A 6-week duration has been used in some previous studies [25], and was chosen to allow sufficient time for structural changes in cortical and trabecular bone to occur. In our previous study [12], we observed decreased metaphyseal trabecular BV/TV after 1 week of tibial compression, despite increased cortical bone formation at the mid-diaphysis. We reasoned that this response may have been transient, and that a 6-week duration would provide an assessment of structural effects at equilibrium.

### In vivo microCT

In vivo microCT was used to assess bone structure at three timepoints (baseline, middle, end) (Figure 3). The baseline scans were performed on the Thurs or Fri before the first loading session; the middle scans were performed on the Tue or Wed in the 3^rd^ week of loading; the end scans were performed on the Mon or Tue after the last loading session. Mice were anesthetized (2–3% isofluorane) and each leg scanned separately (70 kV, 114 µA, medium resolution, 21 µm voxel, 100 msec integration; VivaCT40, Scanco, Brüttisellen, Switzerland). The scanner was calibrated weekly against a hydroxyapative (HA) mineral phantom; density is expressed as mg HA/cm^3^. The leg was positioned parallel to the z-axis of the scanner so that scan slices were in the transverse plane. Diaphyseal scans were obtained over a ∼0.6 mm region located 5.0 mm proximal to the distal tibio-fibular junction, and metaphyseal scans obtained over a ∼2.0 mm region at the proximal metaphysis. To determine cortical parameters, 30 diaphyseal slices (0.63 mm length) were analyzed. The periosteal margin was defined using hand drawn contours, followed by a simple threshold to segment bone from non-bone (sigma = 0, support = 0, lower/upper threshold = 180/1000 = 332 mg HA/cm^3^, peel iteration = 0). Cortical outcomes included total volume inside the periosteal margin (TV; this is the 3D analog to periosteal area in 2D), bone volume (BV; the 3D analog to cortical area in 2D), medullary volume and tissue mineral density (TMD). To determine trabecular parameters, the distal-most slice containing any remnant of growth plate was visualized and 30 slices (0.63 mm length) below this slice were analyzed. The endosteal margin was defined using hand drawn contours followed by thresholding (same parameters as cortical, except peel iteration = 4). Standard outcomes for the metaphysis included trabecular BV/TV, Tb.N, Tb.Th, Tb.Sp, and volumetric BMD. Studies in our lab have shown excellent intra- and inter-operator repeatability using the above protocols (intraclass correlation coefficients >0.90).

### Statistical analysis

Effects of age and loading were assessed by analysis of variance (ANOVA; Statview 5.0, SAS Institute, Cary, NC, USA), followed by Tukey-Kramer multiple comparison tests (2 vs. 4 vs. 7 vs. 12 months) or paired t-tests (loaded vs. non-loaded) if overall ANOVA effects (or interactions) were significant (p<0.05). For baseline controls, effects of age were assessed by one-factor ANOVA. For loaded mice, effects of age and loading were assessed by two-factor ANOVA; in addition, relative data (loaded minus non-loaded; 6-week minus baseline) for microCT outcomes were analyzed by one-factor ANOVA, while temporal changes were assessed by repeated measures ANOVA.

## Supporting Information

Table S1 Primers were either purchased as prescribed sequences from IDT and then validated, or purchased as proven primer sets from Qiagen.
(DOCX)
