# Supplementary material for: Tibial Loading Increases Osteogenic Gene Expression and Cortical Bone Volume in Mature and Middle-Aged Mice
Source: PLoS One. 2012 Apr 13;7(4):e34980. doi: 10.1371/journal.pone.0034980 (PMC3325918; doi:10.1371/journal.pone.0034980)
Supplement: Table S1 — Primers were either purchased as prescribed sequences from IDT and then validated, or purchased as proven primer sets from Qiagen. (DOCX) [file pone.0034980.s001.docx]

**Supplemental Table S1**. Primers were either purchased as prescribed sequences from IDT and then validated, or purchased as proven primer sets from Qiagen.

| Gene | Name | Primer Sequence |  |
| --- | --- | --- | --- |
| *Bmp2* | Bone morphogenetic protein 2 | GGGACCCGCTGTCTTCTAGT | Forward |
|  |  | TCAACTCAAATTCGCTGAGGAC | Reverse |
| *Runx2* | Runt related transcription factor 2 | GGACCGTGGTTACCGTCATG | Forward |
|  |  | GCTCGGCAGAGTAGTTCTCATCA | Reverse |
| *Osx* | Osterix | CCCTTCTCAAGCACCAATGG | Forward |
|  |  | AAGGGTGGGTAGTCATTTGCATA | Reverse |
| *Alp* | Alkaline phosphatase | ACACCAATGTAGCCAAGAATGTCA | Forward |
|  |  | GATTCGGGCAGCGGTTACT | Reverse |
| *Col1a1* | Type I collagen, alpha I | GCTCCTCTTAGGGGCCACT | Forward |
|  |  | CCACGTCTCACCATTGGGG | Reverse |
| *Bsp* | Bone sialoprotein | CCGGCCACGCTACTTTCTT | Forward |
|  |  | GGACTGGAAACCGTTTC | Reverse |
| *Bglap* | Bone gamma-carboxy-glutamic acid-containing protein; osteocalcin | CAGCGGCCCTGAGTCTGA | Forward |
|  |  | GCCGGAGTCTGTTCACTACCTTA | Reverse |
| *Rankl* | Receptor activator of nuclear factor kappa-B ligand | Qiagen Cat. No  QT00147385 |  |
| *Opg* | Osteoprotegerin | Qiagen Cat. No.  QT00106757 |  |
| *Ctsk* | Cathepsin K | AGGCAGCTAAATGCAGAGGGTACA | Forward |
|  |  | AGCTTGCATCGATGGACACAGAGA | Reverse |
| *Cyclo* | Cyclophilin | AGCATACAGGTCCTGGCATC | Forward |
|  |  | TTCACCTTCCCAAAGACCAC | Reverse |
